# Rethinking feature representation and attention mechanisms in intelligent recognition of leaf pests and diseases in wheat

**DOI:** 10.1038/s41598-025-99027-3

**Published:** 2025-05-05

**Authors:** Yuhan Zhang, Dongsheng Liu

**Affiliations:** 1 School of Information Science and Technology, Harbin Institute of Technology (Weihai), Weihai, 264200 China; 2 School of Computer and Communication Engineering, Northeastern University at Qinhuangdao, Qinhuangdao, 066004 China

**Keywords:** Neural network, Prediction model, Big data, Wheat recognition, Disease recognition, Attention mechanism, Feature fusion, Computer science, Information technology, Statistics

## Abstract

Complex pest and disease features appearing during the growth of wheat crops are difficult to capture and can seriously affect the normal growth of wheat crops. The existing methods ignore the full pre-interaction of deep and shallow features, which largely affects the accuracy of identification. To address the above problems and needs, we rethink the feature representation and attention mechanism in intelligent recognition of wheat leaf diseases and pests, and propose a representation and recognition network (RReNet) based on the feature attention mechanism. RReNet captures key information more efficiently by focusing on complex pest and disease characteristics and fusing multi-semantic feature information. In addition, RReNet further enhances the perception of complex disease and pest features by using four layers of detection units and fast IoU loss function, which significantly improves the accuracy and robustness of wheat leaf disease and pest recognition. Tests on a challenging wheat leaf pest and disease dataset with twelve pest and disease types show that RReNet achieves precision, recall and mAP as high as 94.1%, 95.7% and 98.3% respectively. Also, ablation experiments proved the effectiveness of all parts of the proposed method.

## Introduction

Crop pests and diseases limit crop yields and the sustainability of quality and efficient agriculture. Initially, crop pests and diseases detection is mainly carried out by manual methods, the method of transition relies on expert knowledge, and requires a lot of time to complete. The rapid development and improvement of intelligent identification technology provides more advanced technical means for crop pest detection, and crop pest detection has become a technical hotspot in the field of intelligent agriculture today.

In recent years, deep learning-based recognition algorithms for complex diseases and pests of wheat leaves have received more and more attention from researchers. Xu et al.^1^ used a convolutional neural network to extract feature information of leaf pests and diseases in wheat, respectively, and then optimized the basic functions by using residual channel attention blocks to improve the accuracy of classifying diseases and pests of wheat crops. Yang et al.^2^ proposed a lightweight algorithm for leaf pest and disease detection in wheat, which is based on the innovations and improvements made by YOLOv8. Jiang et al.^3^ proposed a model to effectively improve the recognition accuracy of wheat leaf diseases and pests from the perspective of multi-task learning. From the existing recognition methods, the overlapping interference problem existing in wheat leaf disease and pest recognition scenarios is mainly solved by introducing attention module, lightweight improvement, and pre-training knowledge migration to achieve the purpose of improving the recognition accuracy of wheat leaf disease and pest. However, the existing methods ignore the full pre-interaction of deep and shallow features, which greatly affects the accuracy of recognition.

Based on the above problems and needs, we rethink the feature representation and attention mechanism in intelligent detection of wheat leaf pests and propose a representation and recognition network (RReNet) based on the feature attention mechanism.

In summary, the contributions of this paper are as follows:

1. We propose RReNet, which can effectively improve the recognition ability of complex pests and diseases in wheat leaves, and can be effectively monitored during wheat generation, improving the problem of low detection accuracy of existing wheat pest and disease recognition algorithms.
2. We propose a complex pest and disease feature representation module. This module effectively combines the convolutional downsampling module, the convolutional feature extraction group and the channel attention, which effectively improves the representation ability of complex pest and disease features.
3. We propose the pest and disease feature attention mechanism. We fully optimize the deep and shallow feature representations in the complex pest and disease feature representation module, while pre-interacting the deep and shallow features.
4. We add a Transformer^4^ detection unit to the recognition network and introduce a fast IoU. This helped the recognition network to effectively improve the feature representation of complex contextual semantic information of pests and diseases, and made the network converge faster.

## Related work

### Target detector for natural scenes

A target detector in a natural scene usually is comprised of three components, a backbone network composed of deep convolutional neural networks, a neck structure incorporating a multi-scale feature fusion mechanism, and a head designed for targeting the subject.

Since the introduction of AlexNet^5^ in 2012, there has been a proliferation of deep convolutional neural networks. In 2014, the VGG^6^ network was greatly improved from AlexNet by utilizing multiple smaller convolutional kernels instead of large convolutional kernels in the sensory field. ResNet^7^ extends the depth of the convolutional neural network with residual blocks to 101 layers. ResNeXt^8^ proposed depth-separable convolution, and DenseNet^9^ broadened the network structure. darknet^10^ became the most popular backbone network in the field of target detection at one stage.

In terms of multi-scale feature fusion mechanism, Lin et al.‘s^11^ Feature Pyramid Network (FPN), which performs feature extraction by top-down and enables the network to capture target features in different scales of semantic information by fusing multi-scale feature information. Subsequently, Tan et al. improved the feature pyramid network by proposing a bidirectional feature pyramid network (BiFPN^12^) that is able to fuse multiscale features more efficiently in order to improve the performance of EfficientDet on the target detection task. In order to be able to carry out sufficient feature interaction between deep semantic information and shallow semantics to improve the network’s representation of multiscale features, BiFPN adds a bottom-up fusion path to the top-down feature fusion of FPN.

Subsequently, other researchers proposed finer and more complex multi-scale feature fusion mechanisms. Liu et al. proposed Pyramid Attention Feature Pyramid Network (PAFPN^13^), which is also known as Path Aggregation Network (PANet). PANet is an enhanced Feature Pyramid Network (FPN), which further enhances the feature fusion and representation capabilities by introducing an attention mechanism. The design goal of PAFPN is to better utilize multi-scale features to improve the performance of computer vision tasks, especially target detection and segmentation tasks. PAFPN provides an enhanced feature fusion and representation capability by using attention mechanisms, such as Channel Attention and Spatial Attention, to improve the performance of computer vision tasks, especially target detection and segmentation tasks. Spatial Attention), which weight the features. These attention modules adaptively adjust the importance of different features, highlighting key features and suppressing irrelevant or redundant features. Similar to FPN, PAFPN also employs a multi-level feature fusion strategy, where feature fusion is performed step by step from shallow to deep layers. The difference is that PAFPN introduces the attention mechanism in the fusion process of each layer, which makes the features of each layer more flexible and intelligent in fusion, and thus shows better robustness and flexibility in various complex scenarios. The introduction of the attention mechanism enables the network to better adapt to different input features and improve the overall performance. Liu et al. also introduced the concept of Adaptively Spatial Feature Fusion (ASFF^14^), which introduces a flexible spatial fusion mechanism and an adaptive weight learning mechanism to fuse multi-scale feature information more effectively.

The primary focus of the multi-scale feature fusion mechanism discussed above is the integration of features at various levels to enhance the network’s feature representation performance. Nevertheless, these studies overlook the issue of information loss that may occur when features from different levels directly interact with each other.

In terms of anchor frame-based target detectors for natural scenes, they are mainly categorized into one-stage object detectors and two-stage object detectors. The most representative one-stage object detector is the YOLO series^15^. The R-CNN series is the most representative two-stage object detectors. Among them, one-stage target detectors are characterized by real-time and two-stage target detectors are characterized by high network complexity. In terms of one-stage target detectors, the YOLO series has gone through iterations from YOLOv1^16^ to YOLOv9^17^ and has been widely used for its efficient training and inference. The second-stage target detectors have gone through the development from Fast R-CNN^18^, Faster R-CNN^19^ to R-FCN^20^ and are widely used for their high detection accuracy. In addition to anchor-based target detectors, anchor-free detectors are gradually developed, which do not need predefined anchor frames and can predict the center point, size and other attributes of the target directly on the image. Anchor-free detectors have gone through the development from CenterNet^21^, FCOS^22^, CornerNet^23^ to ExtremeNet^24^, and this class of methods has been widely used to simplify the model structure.

One-stage target detectors (Yolo series), two-stage target detectors (Faster R-CNN series), and unanchored frame target detectors (CenterNet series) are the mainstream supervised learning-based target detectors, and each of them has different application scenarios: the Yolo series is suitable for real-time response application scenarios, and the Faster R-CNN series is suitable for scenarios with high accuracy and without real-time fast detection, and the CenterNet series is suitable for scenarios that do not require high detection accuracy but require high detection speed. All of the above methods are applications of supervised learning for target detection in natural scenes. In addition to this, there are researchers who utilize unsupervised learning to achieve target detection in natural scenes.

In addition to target detection methods, image segmentation methods can also achieve the classification and localization of targets in natural scenes. The current research on image segmentation can be based on three methods: FCN^25^, SegNet^26^, and UNet^27^.

The Yolo series may struggle with detecting tiny objects, such as diseases and pests in wheat crops. These small pests and diseases are initially difficult to detect and are often found in shallow features within deep networks. This can result in lower accuracy when trying to recognize diseases and pests in wheat crops. On the other hand, the Faster R-CNN family can improve detection accuracy and adaptability to complex scenes. However, the complex network structure and slower inference speed of Faster R-CNN can hinder its development in the field of wheat crop pest and disease recognition. The CenterNet series, while not as stable and accurate as traditional anchor frame-based detectors in complex scenarios, may struggle due to its direct regression to the bounding box. Unsupervised detection models also lack recognition accuracy and reliability, making them unsuitable for high-accuracy detection in wheat crop pest and disease scenarios. Segmentation models can impact inference speed and may not meet the real-time detection needs in wheat crop pest scenarios. Additionally, existing supervised detection methods often create overly complex models to enhance feature extraction, leading to overfitting and weak generalization when dealing with small sample sizes, limiting their application.

### Detectors in the field of crop pest and disease identification

In the field of crop pest and disease identification, the detection accuracy and detection speed of the identification algorithms play a crucial role in the early screening and intervention of crop pests and diseases, and in order to adapt to that challenge and demand, the relevant researchers have developed a number of specifically optimized algorithms for crop pest and disease identification.

In the area of wheat disease classification, González et al.^28^ contributed to wheat rust resistance and proposed an application of machine learning methods. Goyal et al.^29^ contributed to the detection and classification of wheat leaf pests and diseases by exploring improvements in the architecture of a deep convolutional neural network. Dong et al.^30^ also explored the area of wheat pests and diseases classification by improving the fitness of a classification network in the area of wheat leaf pests and diseases by adding branches between deep convolutional layers. They aimed to improve the fitting ability of the classification network in the field of wheat leaf pests and diseases by adding a branch between the deep convolutional layers, which amplifies the difference between the model output and the actual results, which is often very small. Kukreja et al.^31^ proposed a Mask Scoring RCNN model for wheat aphid disease implementation identification. Sabanci et al.^32^ proposed a novel convolutional-cyclic hybridization network and applied the network to sunn pest-damaged (SPD) wheat kernels, and achieved accurate identification of SPD wheat kernels through a hybrid architecture of AlexNet migration learning and Bidirectional Long Short-Term Memory (BiLSTM).Yao et al.^33^ conducted research in wheat pest classification, improved the CNN, and proposed a novel identification method for winter wheat pests and diseases, which is based on VGG16, on which the attention is used for improvement and the migration learning technique is used to improve the training process, thus effectively improving the classification accuracy of winter wheat pests and diseases.

In addition to the identification and screening techniques for wheat pests and diseases, a considerable number of researchers have contributed to the detection of many crops such as tomato, rice, maize, cassava, etc. Zhang et al.^34^ proposed an improved Faster RCNN algorithm for the detection of powdery mildew, wilt and other diseases in tomato leaves, which uses a deep residual network instead of VGG16 for the image feature extraction to obtain more deeper disease features. Liu et al.^35^ constructed a tomato pest and disease dataset and used image pyramids to optimize YOLOv3 for accurate and fast detection of tomato pest and disease locations and classes. Mbouembe et al.^36^ proposed a tomato detection method based on an improved YOLOv4-tiny model, where they replaced the BottleneckCSP module with a simplified version and then used the Content-Aware Feature Reorganization (CARAFE) module at the neck instead of the traditional up-sampling operator to obtain better high-resolution feature maps. Wang et al.^37^ solved the problem of low effectiveness of insect pest detection in paddy fields based on a lightweight model of YOLOv4, which used MobileNetv2 instead of CSPDarknet53 as the backbone network. Yang et al.^38^ proposed an improved YOLOv8 algorithm Gi-YOLOv8 for rice pest and disease recognition, which adds GAM attention mechanism to the YOLOv8 backbone network. Yang et al.^39^ proposed a novel deep convolutional neural network (CNN)-based method for maize pest and disease detection, and they inserted the CSPResNeXt-50 module for the YOLOv7 model. Tang et al.^40^ introduced transformer encoders into the convolutional neural network architecture to enhance its ability to capture global contextual information. Liu et al.^41^ proposed a transformer encoder based on RPSA (regional pale-shaped self-attention) to capture the global information of the feature map and enhance the local information, so as to organically combine the global and local features of the image for a more comprehensive feature representation. Bai et al.^42^ proposed a lightweight pest identification model based on Transformer and super-resolution sampling techniques.

In the above research in the field of crop pest and disease identification, a variety of optimization algorithms for wheat, tomato, and rice pest and disease detection techniques have emerged, but most of the current algorithms still need to improve their detection accuracy and robustness in complex and variable real crop growth scenarios.

Meanwhile, the above algorithms mainly focus on the replacement of the backbone network, the introduction of attention, and model lightweighting, and there is a lack of enhancement of feature extraction and the introduction of contextual semantic information. Based on this idea, we will focus on the feature representation of complex pests and diseases, the attention of pest features, the fusion network of pest attention features, and the capture of contextual semantic information of pests and diseases, focusing on the enhancement of the recognition ability of complex pests and microscopic pests and diseases.

## Proposed RReNet

### General architecture of RReNet

**Fig. 1:**
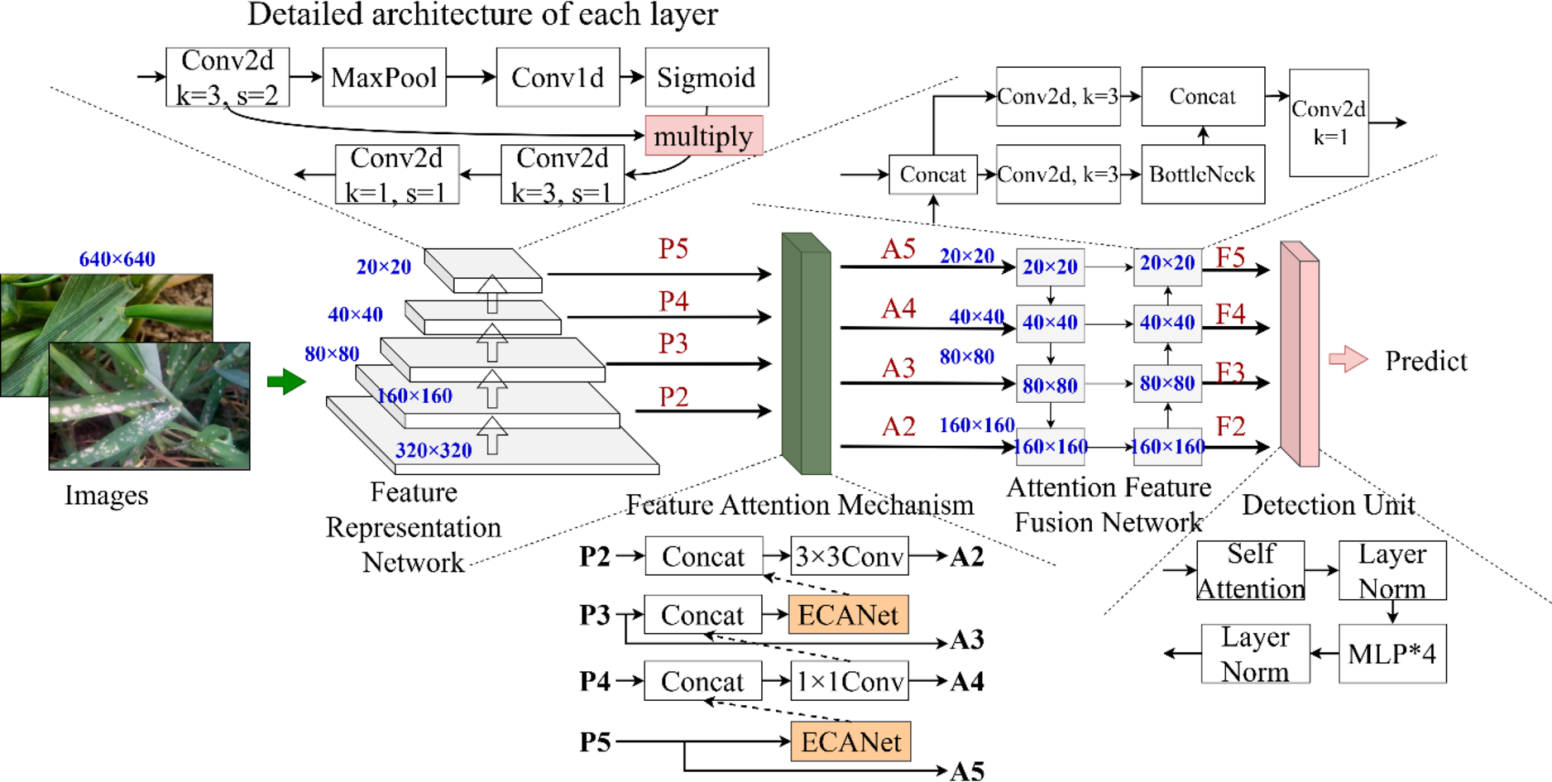
Structure of RReNet, RReNet feeds the input images sequentially into a feature representation network, a feature attention mechanism, a attention feature fusion network and a detection unit for complex disease and pest detection.

RReNet consists of four components: a feature representation network, a feature attention mechanism, an attention feature fusion network, and a detection unit. The feature representation network extracts complex disease and pest features, the feature focusing mechanism pre-interacts four different levels of features from the feature representation network, so as to use the semantic information of the deep-level features to guide the representation of complex disease and pest by the shallow-level features. And the focusing feature fusion network fuses the multi-level features from the feature focusing mechanism and the feature representation network, so as to strengthen the network’s feature representation capability. The detection unit has a first detection unit incorporating a Transformer and three YOLO detection heads. The network architecture is shown in Fig. 1.

Specifically, for the image F input to RReNet, which has been represented by a complex pest feature representation network to represent the image features, the output features of the last four layers are noted as:

1 $${P^F}=\left\{ {{P_2},{P_{3,}}{P_{4,}}{P_5}} \right\}$$

Secondly, this four-layer feature outputs the feature notation after going through the pest and disease feature focus mechanism:

2 $${A^F}=\left\{ {{A_2},{A_3},{A_4},{A_5}} \right\}$$

Again, the output features after going through the attention feature fusion network are noted as:

3 $${F^F}=\left\{ {{F_2},{F_3},{F_4},{F_5}} \right\}$$

### Feature representation network

**Fig. 2:**
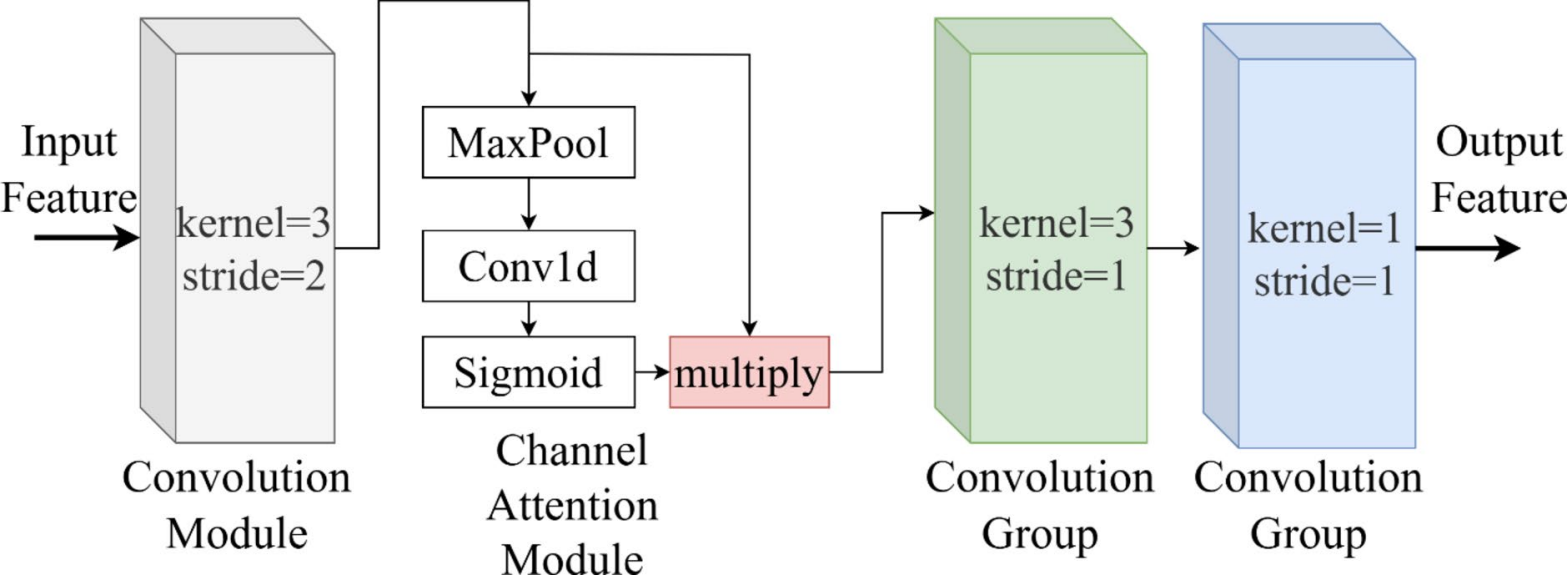
Individual feature extraction and representation layer in complex pest and disease feature representation network.

As shown in Fig. 1, the complex pest and disease feature representation network contains five feature extraction and representation layers. Each feature extraction and representation layer contains four components: A convolutional layer with a step size of 2 performs downsampling of the image, a channel attention mechanism integrates the feature information after downsampling, a convolutional group with a kernel size of 3 accomplishes the task of extracting complex pest and disease features, and a convolutional group with a kernel size of 1 integrates the information. The architecture of each feature representation layer in the complex pest and disease feature representation network is shown in Fig. 2. Specifically, we input the feature map ${F_1}$ into the complex pest and disease feature representation network and denote ${F_5}$ as the output feature map of the first complex pest and disease feature representation layer. Then the generation process of ${F_5}$ is represented as formula (4) (5) (6) (7)

4 $${F_2}=Conv23({F_1})$$

5 $${F_3}={F_2} \cdot Sigmoid(W0(MaxPool({F_2})))$$

6 $${F_4}=\operatorname{Re} LU(BN(Conv3({F_3})))$$

7 $${F_5}=\operatorname{Re} LU(BN(Convl({F_4})))$$

Where Conv23 represents the convolutional module with kernel size 3 in step 2, ReLU represents the activation function, BN represents the normalization, Sigmoid represents the activation function, MaxPool represents the global maximum pooling layer, and Conv3 represents the kernel size 3 in step 1. Convolutional layer with kernel size 3 and step size 1. W0 is a one-dimensional convolution whose represents the convolutional layer with kernel size 1 in step 1.

**Fig. 3:**
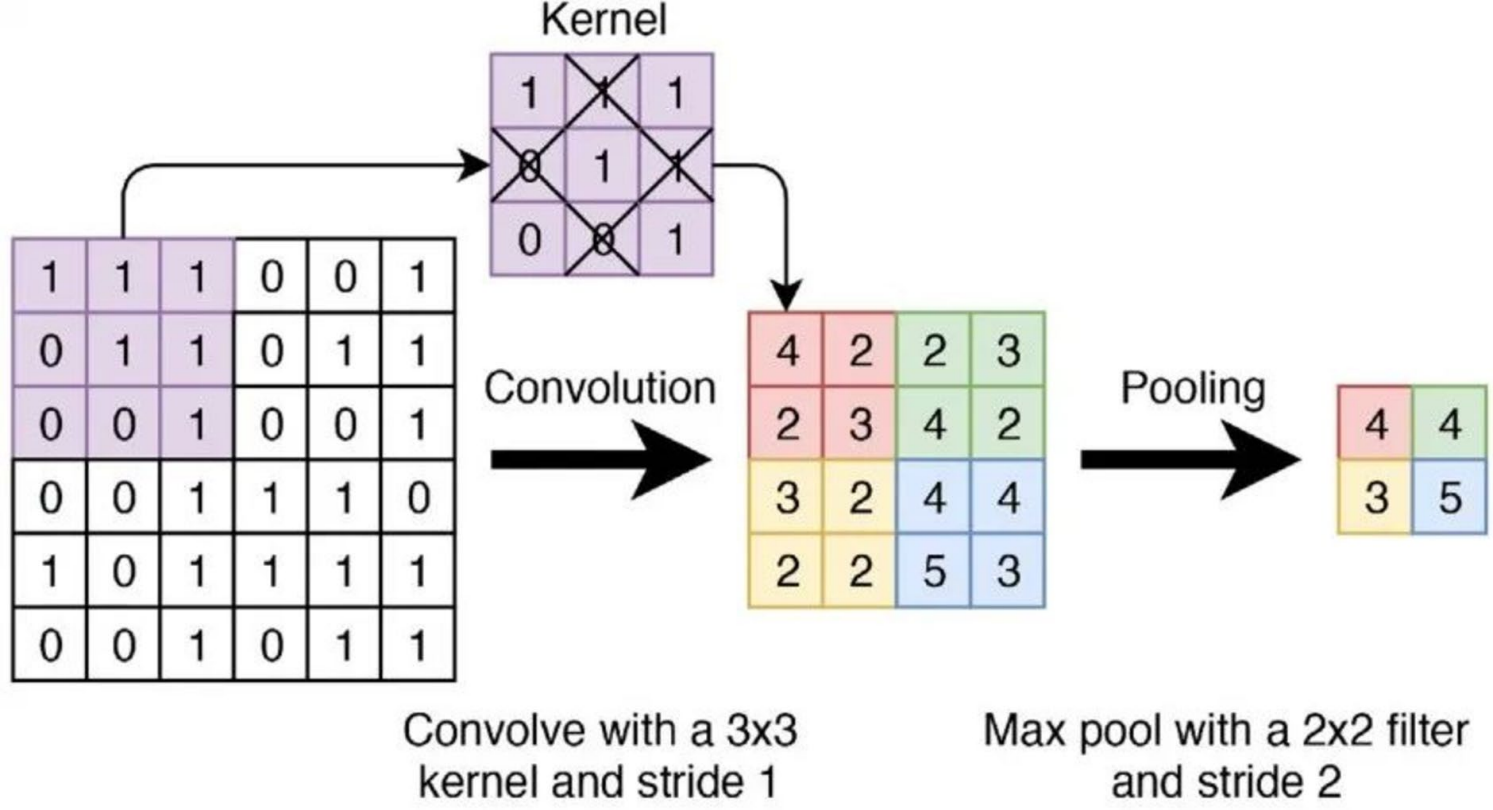
Simplified computational procedure for convolutional and pooling layers in network.

Convolutional layers such as Conv23 and Conv3 and pooling layers such as MaxPool are equivalent to “filter operations” in image processing. Simplify the calculation process in the network, as shown in Fig. 3, the convolutional kernel slides on the input data to calculate the weighted sum, and pooling is used for feature dimensionality reduction, by counting the elements in the pooling window to reduce the size of the data space.

### Feature attention mechanism

We consider pre-interacting the multi-scale features from different layers of the complex pest and disease feature representation network before sending them to the attention feature fusion network for feature fusion, so as to obtain a better feature representation capability when they are fused in the attention feature fusion network.

**Fig. 4:**
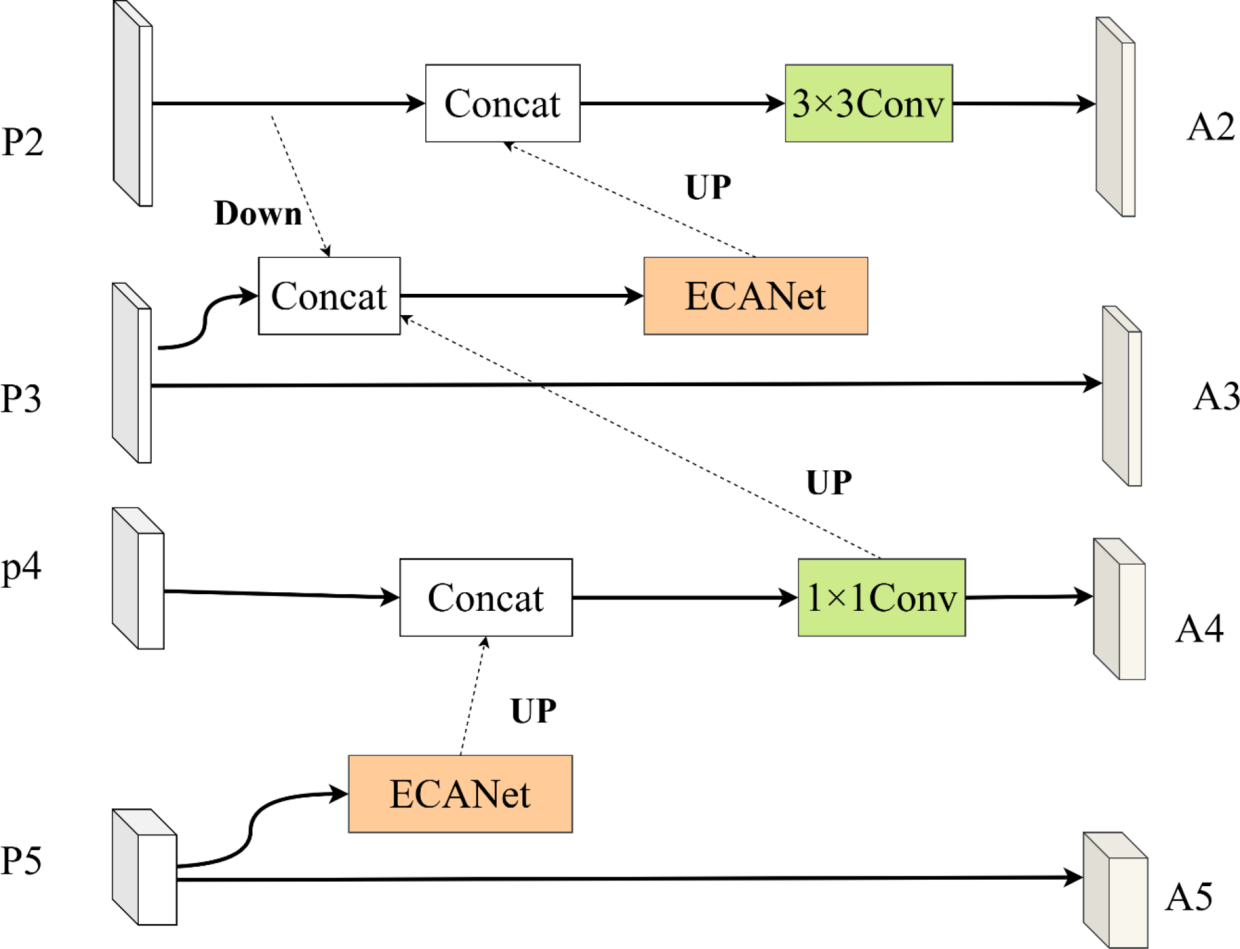
Structure of pest and disease characterization concern mechanism.

Therefore, we propose the pest and disease feature attention mechanism, which is structured as shown in Fig. 4. First, we introduce ECANet^43^ for ${P_5}$ layer, which makes ${P_5}$ layer pay attention to the pest and disease feature information in different channels in time. The layer pays better attention to the channel information. Subsequently, we interact the ${P_5}$ layer with the ${P_4}$ layer, pre-interact the ${P_4}$ layer and the ${P_2}$ layer in the ${P_3}$ layer, and interact the feature information after the interaction with the ${P_2}$ layer, so that the ${P_2}$ layer contains semantic information from the deeper layers, in order to guide the improvement of the recognition performance of the ${P_2}$ layer for the tiny pests and diseases.

This pre-interaction enables shallow features to incorporate global semantic guidance from deeper layers while preserving spatial details, thereby enhancing the network’s sensitivity to tiny pests and complex disease patterns. The detailed process is as follows.

The complex pest and disease representation network outputs four hierarchical features {P_2_, P_3_, P_4_, P_5_}, where F_2_(shallowest layer) captures fine-grained spatial details (e.g., pest edges, lesion textures), and F_5_ (deepest layer) encodes high-level semantic information (e.g., disease categories, contextual relationships).

Each feature P_i_ (i = 2,3,4,5) is first processed by the Efficient Channel Attention Network (ECANet) to generate channel-wise attention weights. This step adaptively enhances critical channels related to pest/disease characteristics while suppressing irrelevant ones.

Deep-to-shallow interaction is performed to propagate semantic information from P_5_ to shallower layers. Specifically:

P_5_ is upsampled to match the resolution of P_4_, then fused with P_4_ through element-wise addition:

8 $${P_4}=Concat({P_4},UpSample(ECANet({P_5})))$$

After pre-interaction, each layer’s feature contains both its original hierarchical information and cross-level contextual cues. For example, P_2_ (shallowest) now embeds semantic hints from P_5_. This pre-interaction ensures that features fed into the subsequent attention fusion network are already semantically enriched and spatially refined, significantly improving the detection of overlapping pests and subtle disease symptoms.

### Feature fusion network attention feature fusion network

**Fig. 5:**
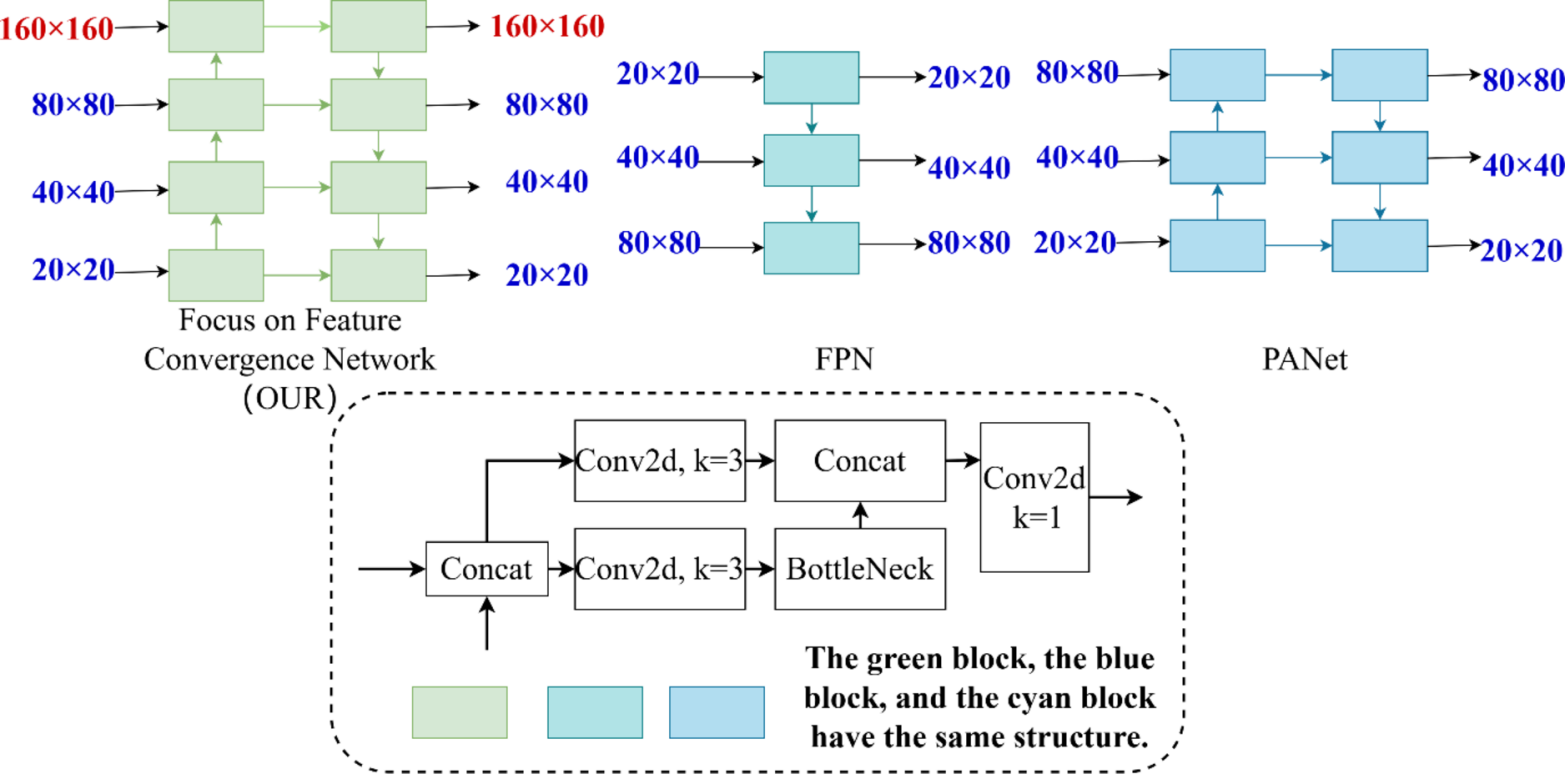
Focus on feature converged network vs. FPN and PANet networks.

The comparison of our proposed key feature fusion network with FPN and PANet is shown in Fig. 5. Among them, our key feature fusion network is significantly different from FPN and PANet: the original PANet structure is three-layered, while our attention feature fusion network is four-layered. Our proposed network contains more complex multi-level feature fusion as well as richer semantic information, which is valuable for the detection of tiny pests in wheat.

### Detection unit

**Fig. 6:**
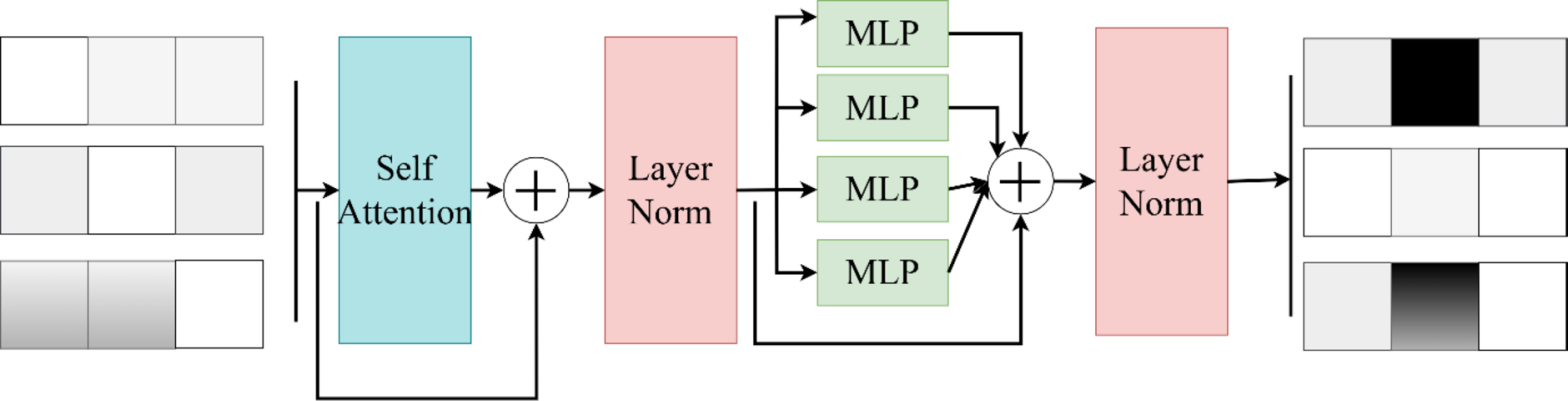
Structure of the transformer block in the first detection unit.

Our detection unit follows the design of YOLO and adds a Transformer detection unit specialized for detecting tiny pests and diseases, which fuses the Transformer block, and its architecture is shown in Fig. 6. This detection unit serves as the first detection unit, which is enhanced by the Transformer block to enhance its feature representation, and at the same time, this module is used to receive the F2 layer (the shallowest layer) of the output of the feature fusion network of interest, which is specialized for the detection of tiny pests and diseases.

The self-attention module in the Transformer module performs feature attention, the multilayer perceptron (MLP) performs feature enhancement extraction, the layer normalization accelerates the convergence of the network, and the DropOut layer is used to prevent the overfitting problem of the complex pest recognition network. By using the Transformer module, the complex pest recognition network RReNet improves its ability to represent contextual semantic information and also enhances the accuracy of RReNet for the recognition of tiny pests and diseases.

### FAST IoU

During the training process, the loss function consists of three parts, one part is the classification loss, one part is the confidence loss, and the other part is the regression loss. Specifically, the loss function is defined as formula (9)

9 $$L=LC+LF+FastIoU$$

where the classification loss is defined as LC and the confidence loss is defined as LF. The regression loss uses our proposed Fast IoU.

Among them, both LC and LF are calculated by the cross-entropy loss function and Focal Loss.

The regression loss function plays an important role in the wheat pest identification task. Recent studies have shown that IoU loss can achieve better results in the recognition task. Currently, CIoU loss^44^ is widely used in detection models such as YOLOv5.

The CIoU loss does not accurately capture the relationship between the aspect ratios of the real frame and the predicted frame, hindering improvements in detection accuracy. To tackle this issue, we have developed a regression loss function called Fast IoU (fast regression intersection and merger ratio loss function). Fast IoU is defined as formula (10)

10 $$FastIoU=1 - IoU+\frac{{{\rho ^2}(b,{b^{gt}})}}{{{C^2}}}+aV+DW$$

Fast IoU contains IoU loss, center point loss, aspect ratio loss and area loss. Among them, IoU loss, center point loss and aspect ratio loss follow CIoU. for area loss. It is calculated in three steps. The first step: calculate the area d1, d2 and d3 of the real frame, the predicted frame and the minimum closure region of the two frames, respectively, and feed d1, d2 and d3 into the Sigmoid function for mapping; the second step, find the ratio of d3 to |d1−d2| to the value of d3/|d1−d2|, which is denoted as W; and the third step, introduce the parameter D that can be trained to update and multiply with W. The third step is to introduce the parameter D that can be trained to update and multiply with W. The first step is to calculate the area of the real frame, the predicted frame, and the minimum closure region of the two frames.

## Experiments

### Experimental setup

This study utilized a 64-bit Ubuntu 18.04 operating system with an NVIDIA A100 model GPU to expedite image processing. The network training employed Mosaic as the data augmentation method, with a BatchSize of 32 and an initial image size of 640 × 640. The model utilized SGD for gradient optimization, with an initial learning rate of 0.01 and annealing cosine for learning rate updates. The model was trained for 500 epochs. The algorithm’s strength and detection accuracy were evaluated using Precision (P), Recall (R), F1-score (F1), and mean Average Precision (mAP).

### Dataset

**Fig. 7:**
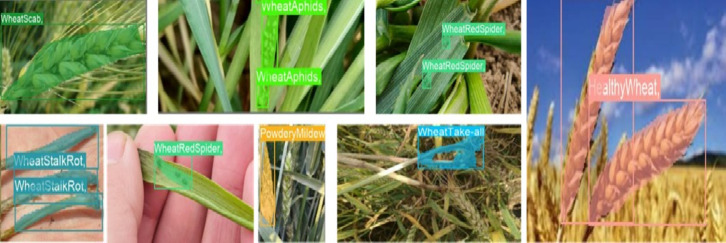
Focus on feature converged networks vs. FPN and PANet networks.

This paper presents experiments conducted on the LWDCD2020 dataset^29^ to confirm the efficacy of the proposed methodology. The LWDCD2020 dataset consists of a total of nearly 7000 close-up images of more obvious wheat diseases, which are classified according to the different types of diseases into 12 categories of common wheat diseases, including healthy wheat, namely, Crown and Root Rot, Leaf Rust, Wheat Loose Smut, Powdery Mildew, Wheat cyst nematode, Wheat scab, Wheat Red Spider, Wheat stalk rot, Wheat Take-all, wheat sharp eyespot, and Wheat Aphids. In this paper, the LWDCD2020 dataset was used to produce its sub-dataset. The sub-dataset was annotated using the Labelimg tool and consists of 12 categories with a total of 900 images depicting wheat pests and diseases. Of these, 720 images were chosen for the training set and 180 images for the test set, known as LWDCD2020Detection. Figure 7. shows the samples of certain categories in the LWDCD2020Detection dataset images and annotations.

### Comparison experiments

In this paper, some SOTA methods (Faster RCNN, CenterNet, YOLOv3^45^, YOLOv4^46^, YOLOv5L^47^, YOLOv7^48^, YOLOv8L^49^, etc.) are selected to compare with the proposed RReNet on the LWDCD2020Detection dataset to validate the generalization ability of the proposed method. Table 1 demonstrates the comparison results of RReNet with the SOTA method on the LWDCD2020Detection dataset.

**Table 1:** Results of comparison experiments on the LWDCD2020Detection dataset.

Method	Param.	GFLOPs	*P*/%	*R*/%	F1/%	mAP/%
YOLOv3	235 M	197.4	87.1	89.3	88.2	90.7
YOLOv4	244 M	123.8	90.2	89.8	90.0	91.5
YOLOv5L	46.3 M	109.7	92.7	92.4	92.5	93.8
YOLOv7	37.7 M	106.4	92.3	91.4	91.8	92.7
YOLOv8L	43.9 M	166.5	93.3	93.7	93.5	94.6
Faster RCNN	315.4 M	224.5	90.8	92.1	91.4	92.4
CenterNet	108.5 M	137.5	93.5	90.8	92.1	92.5
**RReNet (OUR)**	56.7 M	116.3	**94.1**	**95.7**	**94.9**	**98.3**

Significant values are in bold.

Table 1 demonstrates the comparison results of our proposed RReNet method with other SOTA methods on the LWDCD2020Detection dataset using P, R, F1, and mAP. as can be seen from Table 1, our proposed RReNet achieves the optimal result of 98.3% on mAP, which outperforms other SOTA methods. Specifically, it is 6.8%, 4.5%, 5.6%, 3.7%, 5.9%, and 5.8% higher than YOLOv4, YOLOv5L, YOLOv7, YOLOv8L, Faster RCNN, and CenterNet, respectively, and improves by 7.6% compared to the worse YOLOv3 model. Therefore, RReNet can be very effective in wheat pest and disease detection scenarios.

Meanwhile, as can be seen from Table 1, although the Faster RCNN is a two-stage detector with a complex network structure and a huge number of parameters, it does not achieve the expected better results, but rather is inferior to the one-stage models such as YOLOv7 and YOLOv5L, which also shows that it is not the deeper the network hierarchy and the more complicated the network is that can achieve the effective in the complex pest detection scenarios of recognition accuracy, it precisely shows that the dedicated design and specialized application of the network structure is what can enhance the detection of complex pests and diseases in wheat leaves. From Table 1, we can also see that CenterNet, as an anchorless frame detector, achieved 92.5% mAP, which is a small improvement over the second-stage Faster RCNN and a small gap with models such as YOLOv7, but it still has a large gap (5.8% mAP) with our proposed RReNet, which is a greater illustration of the fact that our proposed RReNet is not only more effective than the one-stage YOLO series in the wheat leaf pest detection task, but also achieves better recognition accuracy than two-stage detectors such as Faster RCNN and CenterNet without anchor frame.

RReNet performs well in complex pest and disease feature detection, which is reflected through the following three aspects:

1. RReNet realizes the pre-interaction between deep semantic information and shallow detailed features through the complex pest and disease feature representation module and feature attention mechanism. This design compensates the problem of insufficient interaction between deep and shallow features in existing methods (e.g., YOLO series, Faster RCNN), especially in detecting tiny pests in wheat leaves, the combination of shallow features (edges, textures) and deep semantics (contextual information) significantly improves the feature representation ability.
2. The attention feature fusion network of RReNet contains a four-layer structure (compared with the three layers of FPN and PANet), which supports more complex multi-scale feature fusion, and dynamically adjusts the feature weights through the mechanisms of channel attention (ECA-Net) and spatial attention to inhibit the redundant information, and enhances the ability of sensing tiny targets. In contrast, YOLO series is prone to lose details of shallow features due to high downsampling rate; CenterNet is not stable enough in complex background due to direct regression to bounding box.
3. Through the pest and disease feature attention mechanism, RReNet introduces the ECANet module in the feature interaction process to adaptively strengthen the key channel information, and at the same time enhances the capture of complex contextual semantics by utilizing the long-distance dependency modeling capability of the Transformer detection unit. In contrast, although models such as YOLOv8 introduce attention mechanisms (e.g., GAM), they do not realize the dynamic guidance of cross-layer features. Meanwhile, the Transformer module is embedded in the detection unit to optimize the relationship between local and global features through the self-attention mechanism, which solves the limitation of traditional convolutional networks in long-range dependency modeling, and is especially suitable for the decentralized distribution scenario of leaf diseases.

### Ablation experiment

In order to confirm the efficacy of our proposed modules, we systematically replaced the YOLOv5 baseline with our method. Ablation experiments were carried out on the intricate pest and disease feature representation network (P), pest and disease feature attention mechanism (A), attention feature fusion network (F), and detection unit (D) as shown in Table 2. As we added each component one by one, we noticed a rise in mAP on the LWDCD2020Detection dataset.

**Table 2:** Comprehensive performance experiments on network structures.

Dataset	Baseline	P	A	F	D	mAP/%
LWDCD2020Detection	√	×	×	×	×	93.8
	√	√	×	×	×	95.9 (↑2.1)
	√	√	√	×	×	96.8 (↑3.0)
	√	√	√	√	×	97.7 (↑3.9)
	√	√	√	√	√	97.9 (↑4.1)

We conducted incremental performance tests of our proposed modules, including complex pest and disease feature representation network (P), pest and disease feature focusing mechanism (A), focusing feature fusion network (F), and detection unit (D), based on the baseline. Table 2 illustrates the enhancement in performance achieved by incorporating individual components. Our proposed method demonstrates a significant increase in accuracy compared to the baseline. We first replaced the backbone network with the complex pest and disease feature representation network (P) on top of the baseline, and the accuracy improved to some extent (2.1% mAP improvement for the LWDCD2020Detection dataset). On the basis of replacing the complex pest and disease feature representation network (P), we added the pest and disease feature attention mechanism (A), which achieved a total accuracy improvement of 3.0% mAP, thus improving the recognition ability of RReNet in the face of wheat pests and diseases. On the basis of the above, we proposed the attention feature fusion network (F), and RReNet achieved a total improvement of 3.9% mAP. this also illustrates the effectiveness of the attention feature fusion network (F) in cross-layer information fusion. Subsequently, we proposed the detection unit (D), which improves RReNet by a total of 4.1% mAP.

Meanwhile, in order to better verify the effectiveness of our proposed Fast-IoU loss function, the experiments were conducted with YOLOv5L as the baseline, and the ablation experiments were conducted using DIoU, GIoU, CIoU, EIoU^50^, Alpha-IoU^51^, and our Fast-IoU, respectively, and the results of the experiments are shown in Table 3.

Table 3 presents the comparison results of our Fast-IoU method with other IoU methods on various metrics using the LWDCD2020Detection dataset. These results were obtained by testing the YOLOv5L model. Notably, Fast-IoU outperforms other methods, achieving the highest mAP of 94.1%. It is 0.6%, 0.5%, 0.3%, and 0.8% higher than DIoU, GIoU, CIoU, and EIoU, respectively, and 1.2% higher compared to the worse Alpha-IoU.

It can be obtained from the experiment that Fast-IoU adds a new area loss term (based on the area relationship between the real frame, the predicted frame and the minimum closure area) on the basis of CIoU, and dynamically adjusts the weights through the trainable parameters, so that the loss function optimizes the IoU, the centroid, the aspect ratio, and the area matching at the same time, and it is more comprehensive than the loss of only focusing on a single dimension, such as DIoU, GIoU, and so on. The introduction of the area loss term is particularly suitable for scenarios with large differences in target dimensions in wheat disease detection. For example, the size difference between wheat aphids (tiny) and root rot (larger) is significant, and Fast-IoU more accurately balances the regression weights of different targets through an area-sensitive mechanism. Meanwhile, by introducing a trainable scaling factor (parameter D), Fast-IoU is able to adapt itself to target size changes under different data distributions, which improves the flexibility and generalization ability of bounding box regression.

**Table 3:** Experimental results on the LWDCD2020Detection dataset.

Model	Scale	Method	*P*/%	*R*/%	F1/%	mAP/%
YOLOv5	1.0 (L)	DIoU	92.2	92.7	92.5	93.5
		GIoU	92.6	92.2	92.4	93.6
		CIoU	92.7	92.4	92.5	93.8
		EIoU	92.4	92.1	92.2	93.3
		Alpha-IoU	92.1	91.8	91.9	92.9
		Fast-IoU (OUR)	93.1	93.6	93.3	94.1

## Experiments

This paper addresses the limitations of current methods in identifying wheat pests and diseases by incorporating contextual semantic information and key feature attention. The study delves into feature representation and attention mechanisms in wheat pest and disease identification, introducing RReNet. RReNet comprises four components: a complex pest and disease feature representation network, a pest and disease feature attention mechanism, an attention feature fusion network, and a detection unit. The complex RReNet includes a complex pest and disease feature representation network, a pest and disease feature focusing mechanism, a focusing feature fusion network, and a detection unit. The complex pest and disease feature representation network extracts intricate disease and pest features, while the pest and disease feature focusing mechanism interacts with features at different levels. The focusing feature fusion network combines multi-level features, and the detection unit incorporates a transformer module to focus on long-distance information, enhancing the accuracy and robustness of wheat leaf pest and disease recognition. Tests on a challenging wheat leaf pest dataset with twelve pest types demonstrate that RReNet achieves optimal recognition accuracy compared to the state-of-the-art method. Ablation experiments further validate the effectiveness of the proposed method’s components.

## Data Availability

The datasets used and analysed during the current study available from the corresponding author on reasonable request.
